# Roasting and Cryogenic Grinding Enhance the Antioxidant Property of Sword Beans (*Canavalia gladiata*)

**DOI:** 10.4014/jmb.2003.03069

**Published:** 2020-08-21

**Authors:** Ju-Yeong Jung, Jin-Kyu Rhee

**Affiliations:** Department of Food Science and Engineering, Ewha Womans University, Seoul 03760, Republic of Korea

**Keywords:** Sword bean, *Canavalia gladiata*, roasting, cryogenic grinding, antioxidant

## Abstract

The objective of this study was to optimize the conditions for enhancing the antioxidant properties of sword bean (*Canavalia gladiata*) as a coffee substitute in two processing methods, roasting and grinding. The optimum conditions for removing off-flavor of the bean and maximizing functionality and efficiency were light roasting and cryogenic grinding (< 53 μm). In these conditions, extraction yield was 16.75%, total phenolic content (TPC) was 69.82 ± 0.35 mg gallic acid equivalents/g, and total flavonoid content (TFC) was 168.81 ± 1.64 mg quercetin equivalents/100 g. The antioxidant properties were 77.58 ± 0.27% for DPPH radical scavenging activity and 58.02 ± 0.76 mg Trolox equivalents/g for ABTS radical scavenging activity. The values for TFC and ABTS radical scavenging activity were significantly higher (*p* < 0.05) than in other conditions, and TPC and DPPH radical scavenging activity were second highest in lightly roasted beans, following raw beans. HS-SPME/GCMS analysis confirmed that the amino acids and carbohydrates, which are the main components of sword bean, were condensed into other volatile flavor compounds, such as derivatives of furan, pyrazine, and pyrrole during roasting. Roasted and cryogenically ground (cryo-ground) sword beans showed higher functionality in terms of TFC, DPPH, and ABTS radical scavenging activities compared to those of coffee. Overall results showed that light roasting and cryogenic grinding are the most suitable processing conditions for enhancing the bioactivity of sword beans.

## Introduction

Coffee is one of the most widely consumed non-alcoholic beverages in the world. Caffeine is the most important ingredient of the drink, and a 150 ml cup of coffee contains about 60-120 mg of caffeine. However, caffeine has various effects on the central nervous system and is thought to be the most widely used psychoactive substance. It also increases respiration rate, causes bronchodilation, stimulates lipolysis, and increases diuretic action [1]. As a result of the health concerns mentioned above, many people are replacing caffeinated coffee with decaffeinated coffee as a part of their effort to avoid caffeine; however, decaffeinated coffee also contains a small amount of caffeine [2]. This means that substitution with decaffeinated coffee to eliminate caffeine consumption may not be effective in patients on a caffeine-restricted or abstinence diet. Because of these problems, research continues to find coffee substitutes using a variety of crops. Lee *et al*. [3] studied small, roasted black beans as a coffee substitute for reducing metabolic bone disease in climacteric women. Moreover, in Guatemala in theoasted and ground sword bean seeds were consumed as a coffee-like drink [4]. Likewise, roasted sword bean, called King bean, is consumed as a substitute for coffee in Korea. Since the only such beans commercially available are processed by roasting and grinding, consumers do not have many options in terms of flavor and function.

Sword bean (*Canavalia gladiata*), a perennial vine plant of the bean family, is the largest edible bean plant of the family. It originates in the tropical regions of Southeast Asia and is suitable for cultivation in the southern region of Korea [5]. It has been used as a food and medicinal plant in Asia for thousands of years, and mature seeds are often roasted, ground, and consumed as a drink [6]. Sword bean is rich in phytochemicals like saponin, tannin, flavonoids, terpenoids, and steroids as well as in nutrients such as carbohydrates, proteins, vitamins, and minerals [7, 8]. It also contains urease, hemaglutinine, canavanine, and canavalia gibberellin I and II. The seeds, pods, stems and roots are used in folk remedies that are effective against dysentery, nausea, hemorrhoids, sinusitis, backaches, and obesity. Furthermore, recent studies have shown that sword bean possesses physiological functions such as antioxidant, anti-inflammatory, hematopoietic expansion-improving, hepatoprotective, and anti-angiogenic activities [9-13]. While development and distribution of functional beverages using legumes have been attempted, they have not received much response from consumers due to the peculiar beany flavor [3]. Therefore, to use them as a substitute for coffee beverages, it is necessary to develop a process that can improve the beany flavor of legumes.

Roasting is a process used mainly for coffee, cocoa, and beans in raw material form to produce unique flavors and colors. The roasting process increases the content of water-soluble solids by degradation, synthesis, and condensation reactions and produces brown pigments and aromatic compounds by the Maillard reaction [3, 14]. The structure and components of free amino acids, vitamin E, phytosterols, and lignans change during the roasting process [15, 16]. Song *et al*. [17] researched changes in the physiological properties of mung beans by varying roasting time. As a result, heat treatment for 30 min increased the physiological activities in proportion to roasting time at 110°C. Lee *et al*. [3] reported that heat treatment improves the physiological activity of small black bean (*Rhynchosia nulubilis*) roasted at 110°C and 120°C for 20 min in a study of physicochemical composition and anti-oxidative activities according to roasting temperature. Jeong *et al*. [18] reported that roasting seoritae (black bean) at 110°C for 20 min showed the highest content of isoflavone and antioxidant effect.

In general, grinding as a mechanical process is used to reduce the size of the raw material or to produce a powder. In the milling of food materials, it is often necessary not only to reduce the size of particles, but also to consider sensory properties such as taste, aroma, mouth feel, and color. Food materials are often affected by heat, which can cause deterioration due to heat generated during grinding, resulting in poor quality [19]. In the ambient grinding process, the temperature of the sample rises to 90-95°C, leading to loss of essential oils, aroma, and color, which impairs quality [20]. Cryogenic grinding technology is widely used for materials that are heat-sensitive or oil-/moisture-sensitive or to maintain quality attributes such as volatile oil, flavor, and color. Liquid nitrogen at -196°C absorbs the heat generated during milling to precool the sample and provide the cooling necessary to maintain a low temperature [21].

In this study, we sought to clarify the optimal processing condition for enhancing the functional properties of roasted, ground sword beans for use in beverages as a coffee substitute for people who are unable to ingest caffeine.

## Materials and Methods

### Sample Preparation

Sword beans (*Canavalia gladiata*), cultivated at Jangheung, Jeollanam-do, Korea, were obtained from Gold Farm Food Co. (Korea). The beans were stored at room temperature (25°C) before roasting.

### Roasting

Sword beans were classified into raw bean, light-roasted, medium-roasted, and dark-roasted depending on degree of roasting. Then, 330 g of each bean type was put in a semi-hot air smart roaster (S7, Stronghold, Korea) preheated to 170°C and roasted for different end temperatures as per the degree of roasting. Light, medium, and dark roasting were set based on the time after popping of sword bean seeds. The roasting conditions are shown in Table 1, and the roasted beans were sealed in polyethylene bags and stored at -70°C for use in further experiments.

### Grinding

Roasted sword beans were ground using an air-flow grinder (DCH-500D, Korea). Ambient grinding samples were ground at a scale of 1.0 and 3,000 rpm at room temperature (25°C). Cryogenic grinding samples were ground at a scale of 1.0 and 3,000 rpm at cryogenic conditions (-60°C) using liquid nitrogen. The ground particles of raw bean were sieved with Standard Testing Sieves (Chunggye Sieve Co., Korea) of 60, 100, and 270 mesh. The samples were divided into four size distributions of > 250, 150-250, 53-150, and < 53 μm and were used for measuring extraction yield. To test for functional properties, each roasted, ground powder was sieved with a Standard Testing Sieve of 270 mesh (< 53 μm) to minimize the effects of particle size. All powders were stored at -70°C until use.

### Extraction Efficiency by Particle Size

Stirring extraction was performed following a previous study with simple modifications [10]. Sieved raw sword bean powder (4 g) was extracted with 40 ml of distilled water (DW) and 50% ethanol in a water bath shaker (110 rpm) for 24 h at room temperature (25 ± 1°C). The extract was centrifuged (1,190 g, 25°C, 30 min), and the supernatant was filtered with a syringe filter (0.45 μm diameter, PVDF, Germany). Filtered samples were evaporated, freeze-dried at -88°C in a dryer (FD 8512, IlshinBioBase, Korea), and used for measuring extraction yield according to the following equation:

$$\mathrm{Extraction}\mathrm{yield}(\%)=\frac{\mathrm{Weight}\mathrm{of}\mathrm{powder}\mathrm{after}\mathrm{extraction}}{\mathrm{Weight}\mathrm{of}\mathrm{powder}\mathrm{before}\mathrm{extraction}}\times 100(\%)$$

For measurements of functional properties, 3 g of sword bean powder was extracted with 30 ml of water in a water bath shaker (25 ± 1°C, 110 rpm) for 48 h. After centrifugation (1,190 g, 25°C, 30 min), filtration (0.45 μm diameter, PVDF), and freeze drying (- 88°C, 24 h), the extract was stored at -20°C until use.

### Chromaticity

The chromaticity of roasted, ground bean powders was measured using a colorimeter (Color Quest XE, Hunter Lab, USA). The chromaticity was expressed as values of lightness (L), redness (a), and yellowness (b). The ΔE value, indicating overall difference, was calculated in relation to raw beans by this equation:

$$\Delta E=\sqrt{\Delta L^{2}+\Delta a^{2}+\Delta b^{2}}$$

### Particle Size

The size distribution of roasted, ground bean powders was determined using a laser diffraction particle size analyzer (SALD-2300, Shimadzu, Japan). Sword bean powder was diluted with water and measured with sonication using the light scattering principle. Average particle sizes, D_10_, D_50_, and D_90_, indicate 10%, 50%, and 90%particle size distribution, respectively, and were measured by flow cell type. The percentage of particles in the size ranges of > 250, 150-250, 53-150, and < 53 μm were also measured.

### Scanning Electron Microscope (SEM)

The shape and surface of sword bean powder particles were analyzed using SEM (TM3030Plus, Hitachi, Japan). The images were registered randomly to measure shape and size distribution at specific magnification of 600, with visualization in vacuum conditions and an acceleration voltage of 5.0 kv.

### Determination of Total Phenolic Content (TPC)

The TPC values for extracts were determined using Folin-Ciocalteu’s colorimetric method according to Ramkissoon *et al*. [22] with simple modifications. All dried extracts were diluted to 1 mg/ml with DW. Samples of extracts (0.1 ml) were mixed with 0.4 ml of 7.5% sodium carbonate for 3 min. After 1 hour, 0.5 ml of 10% Folin-Ciocalteu’s phenol reagent was added and absorbance was measured at 750 nm using a spectrophotometer (Spectramax ID3, Molecular Devices, USA). Standards of gallic acid (0-200 mg/g) were used to calibrate the method, and results are expressed as milligrams of gallic acid equivalents per 1 gram of sample (mg GAE/g). Each experiment was performed in triplicate.

### Determination of Total Flavonoid Content (TFC)

The TFC values for extracts were determined using the colorimetric method described previously [23] with simple modifications. Briefly, 0.1 ml of 2% aluminum trichloride (AlCl_3_) in ethanol was mixed with the same volume of samples of extracts (10 mg/ml) for 10 min. The absorbance of the mixtures was measured at 415 nm using a spectrophotometer (Spectramax ID3, Molecular Devices). Quercetin (0-100 mg/g) was used to calibrate the method, and results are expressed as milligrams of quercetin equivalents per 100 grams of sample (mg QE/ 100 g). Each experiment was performed in triplicate.

### Determination of DPPH Radical Scavenging Activity

Radical scavenging activity of the extracts against stable 2,2-diphenyl-2-picrylhydrazyl hydrate (DPPH) was determined according to the method of Blois [24] with some modifications. In brief, a solution of 0.2 mM DPPH in ethanol was prepared. This solution (160 μl) was added to 5 mg/ml samples of extracts (40 μl), and the mixture was allowed to stand at room temperature for 20 min. Absorbance of the mixture was measured at 517 nm using a spectrophotometer (Spectramax ID3, Molecular Devices). Ascorbic acid was used as a positive control. Each assay was performed in triplicate. Free radical scavenging activity was calculated by determining the decrease in absorbance using the following equation:

$$\mathrm{DPPH}\mathrm{radical}\mathrm{scavanging}\mathrm{activity}(\%)=\frac{A_{\mathrm{control}}-A_{\mathrm{sample}}}{A_{\mathrm{control}}}\times 100(\%),$$

where A_control_ was absorbance of the control reaction, and A_sample_ was absorbance in the presence of test sample.

### Determination of ABTS Radical Scavenging Activity

The ability of extracts to scavenge 2,2’-azinobis-(3-ethylbenzthiazoline-6-sulfonic acid) radical cation (ABTS^+•^) was evaluated using the Antioxidant Assay Kit (CS0790, Sigma-Aldrich, USA). The ABTS^+•^ scavenging capacity of sample was expressed in milligrams of Trolox equivalents per 1 gram of sample (mg TE/g) or Trolox equivalent antioxidant capacity (TEAC).

### Headspace Solid-Phase Microextraction (HS-SPME) / Gas Chromatography-Mass Spectroscopy (GC/MS) Analysis

Volatile compounds of sword beans depending on degree of roasting were analyzed using HS-SPME/GC-MS method. A 50/30 μm triple phase of carboxen/polydimethyl-siloxane/divinylbenzene (CAR/PDMS/DVB, Supleco, USA) on an autosampler (CTC CombiPal, CTC Analytics AG, Switzerland) was used for an HS-SPME fiber and for extraction of volatiles in headspace of sample-containing vials. For each HS-SPME analysis, 500 mg of roasted and cryo-ground sword bean was placed in a 20 ml vial. The fiber was preconditioned at 250°C for 10 min in a conditioning station. The vial, tightly capped with a PTFE-silicon septum, was equilibrated for 10 min in the single magnet mixer tray at 50°C and 500 rpm, and then the fiber was exposed to the headspace of sample vial at 50°C for 10 min. For compound desorption, the fiber was placed in the GC injector heated to 230°C for 5 min. The volatile extracts were analyzed on a GC-MS (Agilent 7890B GC connected to an Agilent 7010 Triple Quad MS) equipped with a DM-WAQ capillary column (30 m, 0.25 mm, 0.25 μm phase coating, J&W Scientific). Injection was performed at a split ratio of 15:1 at 230°C for 3 min, with an HS-SPME insert of 0.75 mm ID. The helium carrier gas flow rate was constant at 1 ml/min, and the temperature range in the oven was programmed from 40°C for 1 min to 200°C at 3°C/min to 250°C at 10°C/min for 5 min. The electronic impact ionization method of 70 eV was used. The mass range was 50 to 800 amu at a scanning rate of 5.36 scans/s. The ion source temperature was set at 230°C.

The volatile compounds were identified by comparing their calculated relative retention indexes with those given in the literature and their mass spectra with those in the database (W10N14, Wiley Mass Spectral Data). The relative retention indexes were calculated from the retention times of the linear alkanes (Retention Index Standard, Sigma-Aldrich).

### Comparisons of Antioxidant Activities with Coffee Bean

To compare the functionalities of roasted sword beans with coffee, coffee samples were prepared, and antioxidant properties were evaluated. For comparison using a coffee variety preferred by consumers, the origin and roasting conditions of the sample were set to a condition in which the overall acceptance was high as determined by sensory evaluation in previous studies [25-28].

Brazilian coffee beans were obtained from Woo Sung M. F. Co. (Korea). The beans (500 g) were roasted using a coffee bean roaster (S7, Stronghold). The end temperature and end time were 189 ± 2.08°C and 665 ± 25.3 s, respectively. Roasted coffee beans were ground (-60°C, 3,000 rpm), extracted (90°C, 10 min), filtered (0.45 μm, PVDF), and freeze-dried (-88°C, 24 h) to obtain extract powder as a control. Coffee control was compared with samples of roasted sword beans in terms of total phenolic compounds, total flavonoid compounds, DPPH radical scavenging activity, and ABTS radical scavenging activity.

### Statistical Analysis

All data were presented as mean ± standard deviation (SD). The analysis was performed in triplicate (*n* = 3). One-way analysis of variance with Duncan’s test was performed using SPSS (SPSS, USA). The graph was plotted using SigmaPlot software (version 12.5, USA).

## Results and Discussion

### Physiochemical Properties of Bean and Powder

The values of L (lightness), a (redness), b (yellowness), and ΔE (overall color difference) for each sample were measured using the Hunter color system to compare the color of sword bean according to degree of roasting and grinding condition (Table 2). The L value indicating lightness tended to decrease as degree of roasting increased regardless of grinding condition. The L value of raw, unroasted bean was 82.06-83.89, which was quite high, and decreased from 72.74 (cryo-ground and light-roasted, LC) to 31.86 (cryo-ground and dark-roasted, DC) as degree of roasting increased. However, there was no significant difference by grinding temperature. The values of redness also showed no difference between ambient grinding and cryogenic grinding, increasing from 1.28 (ambient-ground raw bean, RA) to 8.51 (ambient-ground and dark-roasted, DA) with degree of roasting. In the case of yellowness, the highest value of 24.07-24.54 was shown in medium–roasted bean, followed by light-roasted, dark-roasted, and raw bean. The ΔE value, which represents overall color difference in relation to raw bean, was lowest in light-roasted and increased with medium- and dark-roasted bean. This result indicates that lightness and redness have a greater effect on color difference than yellowness. The reason for this change in chromaticity is the Maillard reaction, which changes the taste, flavor, and color of beans during roasting [3].

The particle distribution results for sword beans by degree of roasting and grinding condition are shown in Table 3. The diameter values D10, D50, and D90, representing 10%, 50%, and 90% of the total distribution, respectively, and mean diameter, were investigated. Also, the percentages of beans in the categories of > 250, 150-250, 53-150, and < 53 μm were determined. The average size of particles changed with degree of roasting in both ambient and cryogenic grinding. In ambient grinding, the sizes were significantly larger at 28.23 μm for raw bean and 23.67 μm for light-roasted bean, and there were no significant differences between 19.02 and 18.91 μm for medium- and dark-roasted bean, respectively. Similarly, cryo-ground sword beans showed the largest particle size at 23.72 μm for raw bean, but no significant differences were found between light-, medium-, and dark-roasted bean. The distribution of D10, D50, and D90 diameter values showed that the particles became smaller as the degree of roasting increased. It was possible to infer that the higher the degree of roasting, the lower the moisture content and the more fragile the roasted sword beans become. Warechowska *et al*. [29] investigated whether the increase in average particle size tended with increasing moisture content when grinding wheat with different moisture content.

Generally, cryogenic grinding obtains fine and uniform particles compared to ambient grinding. In a study comparing particles of turmeric with cryogenic and conventional grinding, the mean, standard deviation, minimum, and maximum were smaller with cryogenic grinding [30]. Likewise, when compared in terms of D50 and mean values, the particle size in cryogenic grinding was significantly lower than that in ambient grinding in raw bean and light-roasted bean, and the percentage of sample with size of < 53 μm was significantly higher in cryogenic grinding. In contrast, there were no significant differences of particle size depending on grinding condition for medium- and dark-roasted bean. This is because milling occurred more effectively at cryogenic condition than ambient condition in raw bean and light-roasted bean due to the moisture contained in the sword beans, although the effect of temperature on grinding was not significantly different due to sufficient evaporation of moisture in medium- and dark-roasted bean. The moisture content, crude fat, and total sugar content of sword beans were lower at 8.3, 1.2, and 10.7%, respectively, compared to other food ingredients; thus, it is thought that grinding can be carried out without difficulty in medium- and dark-roasted bean with the moisture removed, even if not at extremely low temperatures [5, 31]. In previous study, grains with low moisture content such as soybean and wheat require less milling time, which means the moisture content of the grains can be controlled to reduce the energy requirements before grinding [32, 33]. Therefore, controlling the degree of roasting or applying cryogenic grinding is beneficial to obtain small and uniformly sized and shaped particles.

**Fig. 1:**
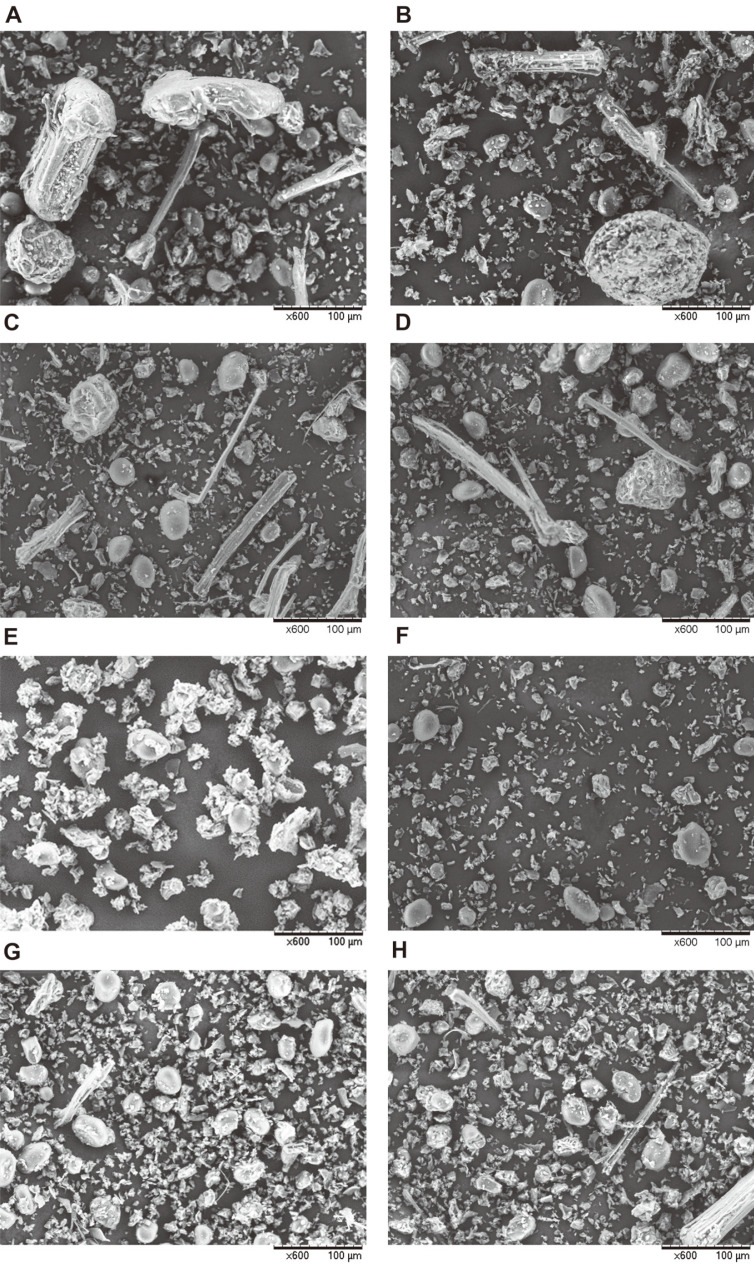
Scanning electron microscope image at 600× magnification of sword beans by degree of roasting and grinding condition are shown. (**A**) RA; (**B**) LA; (**C**) MA; (**D**) DA; (**E**) RC; (**F**) LC; (**G**) MC; (**H**) DC. RA = ambient-ground raw bean; LA = ambient-ground and light-roasted; MA = ambient-ground and medium-roasted; DA = ambient-ground and dark-roasted; RC = cryo-ground raw bean; LC = cryo-ground and light-roasted; MC = cryo-ground and medium-roasted; DC = cryo-ground and dark-roasted.

The shapes of sword bean particles according to degree of roasting and grinding conditions were visualized using SEM as shown in Fig. 1. When ambient grinding was applied, the particles were irregular in shape, large particles were mixed among smaller sizes, and shells that were not crushed were bar-shaped. The size range of these particles was wide (typically 10-100 μm). On the other hand, cryo-ground particles were generally of uniform shape, and distribution of particle size was narrow, typically 10-20 μm. There were no significant differences by degree of roasting. According to these results, the cryogenic grinding method is more effective for grinding sword beans uniformly than is ambient grinding and corresponded with the former results for particle size.

### Extraction Yield

To investigate extraction efficiency for particle size and extraction solvent, cryo-ground, raw sword bean powder was distributed into four ranges of > 250, 150-250, 53-150, and < 53 μm. Extraction was performed by dividing solvent into water, 50% ethanol, and ethanol to observe the extraction yield according to properties of the solvent (Table 4). As a result, the smaller the particles, and the more frequently water was used as a solvent, the higher the extraction yield was. There was significant difference between the extraction yields of ethanol extract in the > 250 μm (0.33%) samples and water extract in the < 53 μm (16.75%) samples. Cho *et al*. [34] showed that the extraction yield of sword beans was highest in water at 17.6%, while the extraction yield in ethanol was very low at 0.1%. Kim *et al*. [31] reported the highest value of 24.8% for hot-water extraction of sword beans and only 2.4% for ethanol extraction, which is very similar to the results of the present research. Therefore, the extracting range for < 53 μm particles with water for solvent is the most efficient, and measurement of functional properties was performed with extracts of roasted sword beans according to extraction conditions.

### Bioactive Compounds

**Fig. 2:**
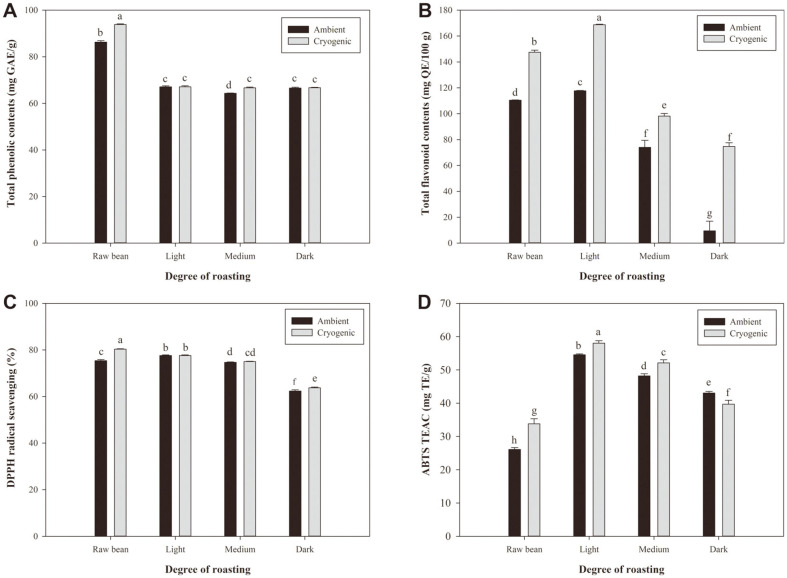
Bioactive properties of sword beans; Raw, light, medium, and dark are degrees of roasting of sword bean, respectively. (**A**) Total phenolic content; (**B**) Total flavonoid content; (**C**) DPPH radical scavenging activity; and (**D**) ABTS TEAC. Values with different alphabetical letters are significantly different at *p* < 0.05 (ANOVA with post-hoc Duncan’s test).

The TPC in terms of GAE of extracts of sword bean by degree of roasting and grinding condition are shown in Fig. 2A. The condition with the highest TPC was raw bean with cryogenic grinding, with a value of 93.84 ± 0.30 mg GAE/g. The second highest value was raw bean with ambient grinding, with a value of 86.30 ± 0.62 mg GAE/g. Cryogenic grinding of raw bean produced 8.03 ± 0.72% higher TPC than ambient grinding. These results indicate that grinding condition has an effect on phenolic content. Moreover, these values are higher than those of a previous study reporting a 57.98 ± 1.19 mg GAE/g DW TPC value of DW extracts of red sword bean coat [35]. Therefore, it was confirmed that extracting a whole bean had higher antioxidant activity than extracting a bean coat. The TPC was significantly decreased by roasting. No significant difference was observed between light-, medium-, and dark-roasted bean. Comparing the differences between cryogenic grinding and ambient grinding, the TPC of cryogenic grinding was higher with medium- and dark-roasted bean, at levels of 3.51% and 0.17%, respectively.

Phenolic compounds are the most common of the various bioactive components in plant foods, and they have high antioxidant activities. In previous studies, the phenolic compounds of sword bean were gallic acid, methyl gallate, monogalloyl hexoside, digalloyl hexoside, digallic acid, trigalloyl hexoside, gallotannin, and protocatechuic acid [10, 35, 36]. These gallic acid derivatives possess various biological functions such as antioxidant activity, antimicrobial effect, anticancer effect, and hepatoprotective effect [37-40]. However, the TPC values decreased after roasting, since phenolic compounds such as gallic acid and gallotannin are rapidly destroyed or converted into other compounds at 105-150°C [41]. Therefore, the phenolic compounds with physiological activities in sword beans are unstable under heat processing.

The TFC of sword beans was converted into quercetin equivalent (mg QE/100 g) according to degree of roasting and grinding conditions (Fig. 2B). Ambient and cryogenic grinding showed the highest TFC values of 117.76 ± 0.23 and 168.81 ± 1.64 mg QE/100 g, respectively, in light-roasted bean. In ambient grinding, the values d ecreased in the order of 110.50 ± 0.22, 73.99 ± 5.51, and 9.52 ± 7.43 mg QE/100 g in raw, medium-, and dark-roasted bean, while those for cryogenic grinding were 147.51 ± 1.64, 98.16 ± 2.02, and 74.69 ± 2.87 mg QE/100 g, respectively. This indicates that cryogenic grinding produced significantly higher TFC than ambient grinding under all conditions. The TFC of cryogenic grinding was 25.08, 30.24, 24.68, and 87.44% higher than that of ambient grinding in raw, light-, medium-, and dark-roasted bean, respectively.

Flavonoids obtained as secondary metabolites of plants have anticancer and anti-cardioprotective effects through antioxidant, anti-allergic, anti-inflammatory, antimicrobial, and various other mechanisms of luteolin, tangeritin and catechins (catechin, epicatechin, epicatechin gallate, and epigallocatechin gallate) [42]. The flavonoid compounds contained in sword bean are isoflavones, catechin, epicatechin, robinin, kaempferol, canavalioside, and acylated flavonol glycosides (gladiatosides A1, A2, A3, B1, B3, C1, and C2). In particular, the amounts of catechin and epicatechin are 14.4 ± 1.13 and 25.8 ± 1.21 mg/100 g DW, respectively [42-44]. In this research, the TFC value increased in light-roasted bean and decreased rapidly in medium- and dark-roasted bean. The flavonoid content increased under heat treatment due to synthesis into other polyphenolic compounds, whereas some compounds such as catechin and epicatechin are denatured or destroyed when heated at high temperature over 180°C for a long time. In a previous study, total catechin in grape seeds heated at 100°C for 60 min (3.18 mg/ml) or heated at 150°C for 40 min (3.75 mg/ml) was higher than in a non-heated control (2.32 mg/ml) [45]. Moreover, when comparing the contents of catechin, epicatechin, gallocatechin, epigallocatechin, epicatechin gallate, catechin gallate, epigallocatechin gallate, and gallocatechin gallate of heat-processed green tea, the amounts of catechin, gallocatechin, and gallocatechin gallate increased significantly after heat treatment (steaming or roasting, at 160-230°C for 10-12 min), but flavonols in the heated tea leaves decreased when fixed at high temperature [46]. These results agree with the present results that the value of TFC increased under proper heat-processing of sword bean but decreased at high temperature.

### Antioxidant Properties

The DPPH radical scavenging activity of sword bean extracts by degree of roasting and grinding conditions is shown in Fig. 2C. The result of DPPH assay showed the highest radical scavenging activity with raw bean and cryogenic grinding (80.32 ± 0.20%). The DPPH scavenging ability of raw bean and ambient grinding conditions was 75.38 ± 0.48%, which was about 6.15% lower than that of cryogenic grinding. The second highest value was 77.61% and 77.58 in the ambient and cryogenic grinding conditions of light-roasted bean, respectively, and there were no significant differences (0.04%). The ability to scavenge DPPH radical was decreased in order of medium-and dark-roasted bean, and cryogenic grinding had slightly higher values by 0.42 and 2.17% than ambient grinding. Therefore, DPPH radical scavenging activity was most effective in sword bean when applying raw bean to light-roasting and cryogenic grinding condition.

The method to determine antioxidant capacity, such as DPPH assay, provides broad information on the antioxidants present in biological samples, taking into account addition and synergistic effects of all bioactive substances rather than the effect of a single compound, and it is useful to study the potential of antioxidants for oxidative stress-mediated diseases [47, 48]. Although it is difficult to directly identify the components that influence the DPPH radical scavenging activity of sword beans, compared with the previous results for TPC and TFC, the antioxidants of sword bean tend to be destroyed when applying more than the medium roasting (171°C, 516 s).

The results of ABTS radical scavenging activity by degree of roasting and grinding conditions are shown in Fig. 2D. The highest activity was shown in light roasting and cryogenic grinding, and the TEAC value of this condition was 58.02 ± 0.76 mg TE/g. The raw bean showed the lowest values of 26.08, and 33.80 mg TE/g in ambient and cryogenic grinding, respectively. The value was maximum in light roasting and decreased in order of medium and dark roasting (52.12-39.68 mg TE/g). The difference between ambient and cryogenic grinding was highest in raw bean (22.74 ± 3.02%), and the TEAC value of cryogenic grinding was significantly higher by 5.99 and 7.54% than that of ambient grinding in light- and medium-roasted bean, respectively. However, in dark-roasted bean, ambient grinding was 8.54% higher than cryogenic grinding. In the case of dark roasting, it is thought that the advantage of cryogenic grinding, which prevents loss of thermolabile compounds by minimizing heat generation during milling, was not achieved due to loss of antioxidant components of sword bean in high-temperature treatment.

Floegel *et al*. [49] reported that the antioxidant properties determined by ABTS assay were strongly correlated with the ORAC of the USDA database and phenolic and flavonoid contents in 50 antioxidant-rich foods of the US diet. The results suggest that the ABTS assay is superior to the DPPH assay when applied to various plant foods containing lipophilic, hydrophilic, and high-pigmented antioxidant components. Our study found that the antioxidant capacity of sword bean was enhanced by proper heat treatment and light roasting (163°C, 442 s). This finding is consistent with TFC results which were shown in our study, and it seems that appropriate roasting improves the antioxidant properties by producing flavonoids.

The significance level (*p* < 0.05) of the TPC, TFC, DPPH radical scavenging activity and ABTS TEAC values in extracts of roasted sword bean are shown in Table 5. Overall, the anti-oxidative activity of sword bean was most effective for raw bean with light roasting, followed by cryogenic grinding. However, considering previous research showing that the ABTS assay is more correlated with the antioxidant activity of plant foods than the DPPH assay, it seems to be more appropriate for applying light roasting to improve antioxidant activity. In addition, the size range of < 53μm, which has the highest extraction yield, was larger in cryogenic grinding. Therefore, light roasting and cryogenic grinding are the optimal conditions for increasing efficiency and functionality of sword bean.

In previous study [3], it was determined that the values for TPC, TFC, DPPH radical scavenging effect and ABTS radical scavenging effect were highest at 120°C when the antioxidant activities were measured after roasting small black beans raw, at 90, 100, 110, and 120°C. The mung bean also showed the highest DPPH and ABTS radical scavenging activity when treated at 110°C for 30 min, among 0, 10, 20, and 30 min [17]. These results correlate with the increased anti-oxidative effect of sword bean under light roasting condition heat-treated for about 442 s at 163°C, considering the large seed size of sword bean compared to that of small black bean and mung bean. These results are consistent with studies showing that antioxidant activity is improved by increase of physiologically active substances due to various chemical changes resulting from heat treatment of food [3, 17, 18, 50]. Therefore, it is possible to increase antioxidant activity by applying the roasting process to sword bean and to remove the off-flavor of raw sword bean.

In addition, cryogenic grinding had more effect on antioxidant activity in raw bean; however, when the roasting process was applied, the antioxidative effect of ambient-ground sword bean was similar to that of cryo-ground samples. Considering economics, it is significant that the enhancement of antioxidant effect through roasting process is advantageous compared to application of cryogenic grinding.

### HS-SPME/GC-MS Analysis

**Fig. 3:**
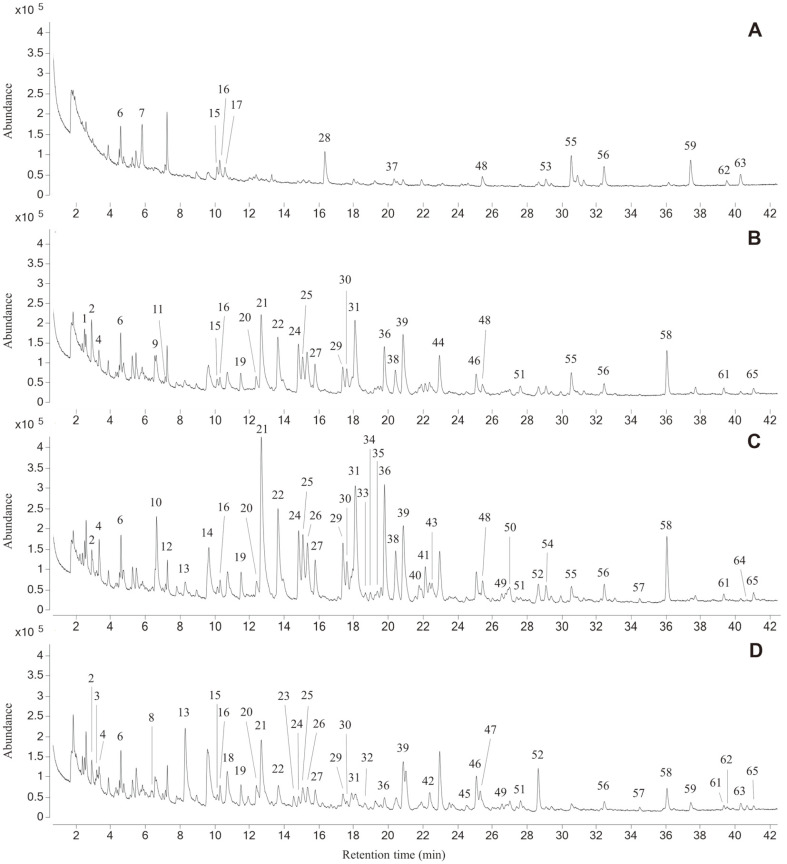
Total ionic chromatograms of volatiles in cryo-ground sword bean obtained by HS-SPME/GC-MS analysis. Numbers correspond to the list in Table 6. (**A**) Raw bean; (**B**) Light roasting; (**C**) Medium roasting; (**D**) Dark roasting.

HS-SPME/GC-MS analysis was performed to identify the volatile components produced during roasting of sword bean (Fig. 3). The relative proportions of each component of the sample, calculated by dividing the peak area by the total peak in the total ion chromatogram obtained by GC-MS, are shown in Table 6. Sixty-five volatile compounds depending on the roasting conditions were identified by GC-MS. Raw bean was identified to contain a total of 14 compounds, whereas 31 compounds were present with light–roasted bean, 42 compounds with medium-roasted, and 31 compounds with dark-roasted bean.

The volatile components produced by roasting of sword beans were identified as furans, pyrazines, pyridines, and pyrroles. Furfural derivatives, such as furfural and furfuryl alcohol, were reported to be sweet and bread-like with a caramel-like flavor [51]. A previous study found that these compounds were formed from the reaction between sugars and amino acids at high temperatures above 98°C [52]. Though this result suggests a composition of various sugars and amino acids in sword beans, ^1^H NMR analysis in this study (data not shown) showed formation of furans during roasting. Moreover, the nitrogen-containing heterocyclic compounds, including pyrazines, pyridines, and pyrroles, are major flavors in coffee [53]. They are mainly produced through the Maillard reaction and are known to provide roasted/toasted flavor in heat-treated foods or drinks [54]. Alkyl-pyrazines identified in roasted sword beans were investigated in the 1970s as compounds involved in browning reaction of sugars and amino acids [55]. Pyrazines in ground roast coffee and coffee brew associated with roasted/burnt, earthy/musty, and woody/papery flavors have been published through studies about correlation between key odorants and flavors in coffee by GC-MS and principal component analysis [56-58]. In this study, different types and amounts of pyrazine were detected in different roasting conditions and were most abundant with medium roasting. Pyridine has a pungent odor and was first reported in coffee in 1946 to produce a pleasant burnt/smoky flavor in extreme dilution [51, 58]. In this study, the highest amount of pyridine was detected in light-to-medium roasting and decreased in dark roasting.

Volatile chemicals have an important influence on taste and preference for heat-treated foods and beverages. In addition, the specific conditions of heat treatment play an important role in the final flavor of food and beverage. The present study found that the roasting conditions significantly changed the concentrations of certain volatile compounds in sword beans. Flavor components such as furans and furfural derivatives, which form from compounds of raw sword beans such as sugars and lipids, were yielded in relatively high concentrations under dark roasting. Otherwise, compounds like pyridines and pyrazines, which form from the Maillard reaction between sugars and amino acids, were more abundant in light-to-medium-roasted sword beans. These results show that roasting of sword beans produces components similar to the main volatile components found in coffee [51,53,56-58]. Compared with the identified flavor compounds and previous results of functional properties in this study, light roasting is appropriate for sword bean drink as a coffee substitute.

### Comparison of Antioxidant Activities with Coffee Bean

**Fig. 4:**
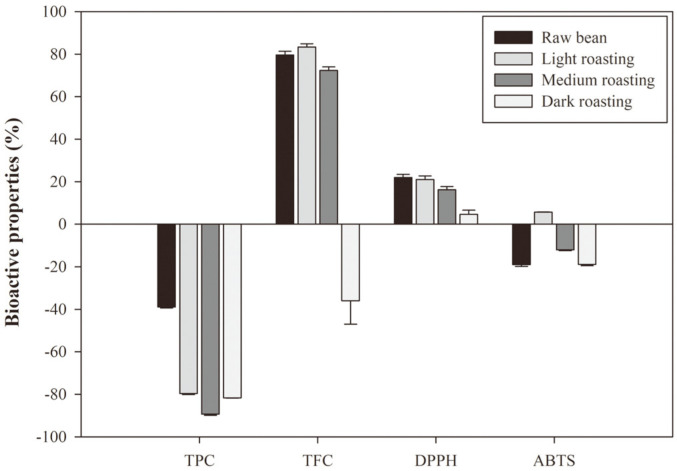
Difference in bioactive properties of cryo-ground sword beans and control. Control = roasted coffee bean; TPC = total phenolic content; TFC = total flavonoid content; DPPH = DPPH radical scavenging activity; and ABTS = ABTS TEAC. Values with different alphabetical letters are significantly different at *p* < 0.05 (ANOVA with post-hoc Duncan’s test).

To investigate the difference of antioxidant activity between coffee and sword bean, a comparative experiment was performed by setting the roasted coffee extract as a control. The results are shown in Fig. 4. The TPC value was highest in the control, and the values were decreased in the order of raw bean, light-roasted, dark-roasted, and medium-roasted bean, and they were 38.94, 79.67, 81.68, and 89.38% lower than the control, respectively. The TFC was significantly higher in light and medium roasting compared to the control. Light roasting showed the largest difference (83.32%) and decreased in the order of raw bean (79.67%) and medium-roasted bean (72.33%). Dark roasting showed 8.82% lower TFC value than the control. The DPPH radical scavenging activity was significantly higher in all roasting conditions than in the control. Raw bean and light roasting showed the highest differences of 21.00-21.89% from the control, and there was no significance difference between the two conditions. Medium and dark roasting were higher for 16.13% and 4.59% than control, respectively. The results were slightly different for ABTS radical scavenging activity. Only light roasting showed a higher value at 5.65%than the control and showed lower activities by 11.97, 18.85, and 18.90% in order of medium roasting, dark roasting, and raw bean.

In conclusion, the optimum conditions to remove off-flavor of sword bean and maximize the functionality were light roasting and cryogenic grinding (<53 μm). The results of this study suggest that the sword bean improves in physiological activity by proper heat processing. Moreover, our study establishes the processing conditions (light roasting and cryogenic grinding) to maximize the functional properties of sword bean. The potential of sword bean drink as a non-caffeine coffee substitute was confirmed by optimizing the processing conditions and this result is expected to provide coffee-flavored, functional beverage products for people who are not able to consume caffeine.

## Supplemental Materials



Supplementary data for this paper are available on-line only at http://jmb.or.kr.

## Figures and Tables

**Table 1:** Roasting conditions of sword beans.

Degree of roasting	End point^a^ (℃)	Holding time^b^ (s)	T.P^c^ (℃)	Time after T.P^d^ (s)
Raw bean	-	-	-	-
Light	163	442.00 ± 7.81^e^	140.33 ± 6.51	370.00 ± 7.81
Medium	171	515.67 ± 4.04	141.33 ± 2.31	428.33 ± 22.81
Dark	186	762.33 ± 5.69	143.33 ± 3.51	644.00 ± 15.88

^a^The temperature when sword bean is released from roasting drum.

^b^Roasting time of sword bean from start to finish.

^c^Turning point, meaning the lowest temperature after the roasting has started.

^d^Time after turning point, meaning the holding time after turning point.

^e^All processes were repeated three times. Values are mean ± SD (*n* = 3).

**Table 2:** Hunter’s color value of sword bean powders by roasting and grinding condition.

Sample	L	a	b	ΔE
RA	83.89 ± 0.01^8^	1.28 ± 0.01^1^	7.97 ± 0.01^1^	-
LA	71.52 ± 0.00^5^	4.73 ± 0.03^4^	20.86 ± 0.02^5^	18.19 ± 0.01^2^
MA	54.55 ± 0.01^3^	7.76 ± 0.04^6^	24.07 ± 0.01^6^	34.08 ± 0.02^4^
DA	34.22 ± 0.02^2^	8.51 ± 0.02^7^	17.73 ± 0.05^3^	51.13 ± 0.01^5^

RC	82.06 ± 0.01^7^	1.91 ± 0.01^2^	9.16 ± 0.02^2^	-
LC	72.74 ± 0.01^6^	4.46 ± 0.01^3^	20.92 ± 0.01^5^	15.22 ± 0.01^1^
MC	57.71 ± 0.02^4^	7.56 ± 0.03^5^	24.54 ± 0.03^7^	29.35 ± 0.02^3^
DC	31.86 ± 0.02^1^	8.48 ± 0.05^7^	18.28 ± 0.08^4^	51.44 ± 0.04^6^

Hunter color value, L: lightness (0=black, 100=white), a: red/green (+; red, -; green), b: yellow/blue (+; yellow, -; blue), ΔE: overall color difference [(ΔL^2^+ Δa^2^+ Δb^2^)^1/2^].

RA = ambient-ground raw bean; LA = ambient-ground and light-roasted; MA = ambient-ground and medium-roasted; DA = ambient-ground and dark-roasted; RC = cryogenic-ground raw bean; LC = cryogenic-ground and light-roasted; MC = cryogenic-ground and medium-roasted; DC = cryo-ground and dark-roasted.

All processes were repeated three times. Values are mean ± SD (*n* = 3).

^1-8^Values with different subscript numbers in the same row are significantly different at *p* < 0.05 (ANOVA with post-hoc Duncan’s test).

**Table 3:** Particle size distribution of ground sword beans.

Sample	D10 (μm)^a^	D50 (μm)^a^	D90 (μm)^a^	Mean D (μm)	> 250 μm(%)^b^	150 - 250 μm (%)^b^	53 - 150 μm (%)^b^	< 53 μm(%)^b^
RA	6.21 ± 0.02^7^	30.79 ± 0.11^4^	114.36 ± 1.57^6^	28.29 ± 0.16^3^	0.35 ± 0.08	4.91 ± 0.24	21.15 ± 0.36	73.59 ± 0.27^3^
LA	4.23 ± 0.01^4^	28.18 ± 0.15^3^	112.27 ± 0.85^6^	23.67 ± 0.09^2^	0.62 ± 0.06	5.16 ± 0.11	19.80 ± 0.10	74.43 ± 0.25^3^
MA	3.51 ± 0.10^2^	22.34 ± 0.90^1^	90.79 ± 6.96^34^	19.02 ± 0.57^1^	0.05 ± 0.02	2.66 ± 0.78	17.68 ± 0.36	79.61 ± 1.13^1^
DA	3.96 ± 0.05^3^	21.67 ± 0.25^1^	78.62 ± 3.45^1^	18.91 ± 0.34^1^	0.03 ± 0.02	1.82 ± 0.54	14.77 ± 0.22	80.72 ± 0.63^1^

RC	5.41 ± 0.35^5^	25.26 ± 0.09^2^	104.14 ± 2.23^5^	23.72 ± 0.36^2^	0.06 ± 0.01	3.70 ± 0.18	19.27 ± 0.76	76.97 ± 0.91^2^
LC	3.67 ± 0.14^2^	21.49 ± 1.69^1^	92.16 ± 0.98^4^	19.14 ± 0.93^1^	0.21 ± 0.15	3.04 ± 0.73	16.69 ± 1.77	79.91 ± 1.19^1^
MC	3.57 ± 0.01^2^	22.62 ± 0.07^1^	85.25 ± 5.25^23^	18.94 ± 0.29^1^	0.05 ± 0.00	2.35 ± 0.25	16.61 ± 1.03	81.00 ± 1.28^1^
DC	3.24 ± 0.02^1^	24.27 ± 0.23^2^	83.97 ± 18.99^12^	18.99 ± 0.13^1^	0.00 ± 0.00	0.91 ± 0.06	20.14 ± 0.26	78.95 ± 0.29^1^

All processes were repeated three times. Values are mean ± SD (*n* = 3).

RA = ambient-ground raw bean; LA = ambient-ground and light-roasted; MA = ambient-ground and medium-roasted; DA = ambient-ground and dark-roasted; RC = cryo-ground raw bean; LC = cryo-ground and light-roasted; MC = cryo-ground and medium-roasted; DC = cryo-ground and dark-roasted.

^a^Diameter (D)10, D50, and D90 = 10%, 50% and 90% of particle size distribution, respectively. ^b^The percentage of particles in the size ranges of > 250, 150-250, 53-150, and < 53 μm.

^1-7^Values with different subscript numbers in the same row are significantly different at *p* < 0.05 (ANOVA with post-hoc Duncan’s test).

**Table 4:** Extraction yield of sword bean powder by particle size and solvent.

Particle size (μm)	Solvent	Extraction yield (%)
> 250	Water	3.00 ± 0.30^5^
	50% Ethanol	1.32 ± 0.17^6^
	Ethanol	0.47 ± 0.12^7^
150 - 250	Water	3.45 ± 0.31^5^
	50% Ethanol	3.04 ± 0.19^5^
	Ethanol	0.53 ± 0.25^7^
53 - 150	Water	9.70 ± 0.18^2^
	50% Ethanol	5.92 ± 0.32^4^
	Ethanol	1.66 ± 0.13^6^
< 53	Water	17.20 ± 0.51^1^
	50% Ethanol	8.07 ± 0.55^3^
	Ethanol	1.49 ± 0.05^5^

All processes were repeated three times. Values are mean ± SD (*n* = 3).

^1-7^Values with different subscript numbers are significantly different at *p* < 0.05 (ANOVA with post-hoc Duncan’s test).

**Table 5:** Total polyphenol content (TPC), total flavonoid content (TFC), DPPH radical scavenging activity, and ABTS TEAC of sword bean.

Grinding	Roasting	TPC (mg GAE/g)	TFC (mg QE/100 g)	DPPH (%)	ABTS (mg TE/g)
Ambient	Raw	86.30 ± 0.62^2^	110.50 ± 0.22^4^	75.38 ± 0.48^3^	26.08 ± 0.57^8^
	Light	67.11 ± 0.41^3^	117.76 ± 0.23^3^	77.61 ± 0.27^2^	54.54 ± 0.28^2^
	Medium	64.31 ± 0.16^4^	73.99 ± 5.51^6^	74.66 ± 0.19^4^	48.18 ± 0.67^4^
	Dark	66.60 ± 0.34^3^	9.52 ± 7.43^7^	62.33 ± 0.54^6^	43.04 ± 0.49^5^
Cryogenic	Raw	93.84 ± 0.30^1^	147.51 ± 1.64^2^	80.32 ± 0.20^1^	33.80 ± 1.53^7^
	Light	67.07 ± 0.48^3^	168.81 ± 1.64^1^	77.58 ± 0.27^2^	58.02 ± 0.76^1^
	Medium	66.65 ± 0.33^3^	98.16 ± 2.02^5^	74.98 ± 0.16^34^	52.12 ± 0.92^3^
	Dark	66.71 ± 0.20^3^	74.69 ± 2.87^6^	63.71 ± 0.31^5^	39.68 ± 1.22^6^

All processes were repeated three times. Values are mean ± SD (*n* = 3).

^1-8^Values with different subscript numbers in the same row are significantly different at *p* < 0.05 (ANOVA with post-hoc Duncan’s test).

**Table 6:** Identification and relative percentage peak areas of volatile compounds of cryo-ground sword bean.

No.	Compounds	R.T^a^ (min)	GC peak area %^b^			

			Raw bean	Light roasting	Medium roasting	Dark roasting
1	Propanal	2.5		0.89		
2	Furan, 2-methyl-	2.9		2.28	0.90	1.15
3	Pyrazolidine	3.1				0.58
4	Pentanal	3.3		0.82	1.04	1.18
5	Pivalaldehyde	4.3				0.49
6	Decane	4.6	4.19	2.02	1.81	2.22
7	*tert*-Hexadecanethiol	5.8	5.20			
8	Disulfide, dimethyl	6.3				0.56
9	Hexanol-4-d2	6.5		1.06		
10	Butane, 1-isocyano-	6.6			4.44	
11	1-Dodecene	7.1		0.24		
12	1,2,3,5-tetramethylcyclohexane (1*r*,2*c*,3*c*,5*t*)	7.2			0.43	
13	1-Methylpyrrole	8.3			0.55	7.64
14	Pyridine	9.6			8.04	
15	Limonene	10.1	1.30	0.49	0.24	0.21
16	Decane, 2-methyl-	10.3	2.13	0.65	0.54	0.90
17	1-Pentanol	10.6	1.03			
18	Pyrazine	10.7				2.56
19	Furan, 2-pentyl-	11.5		1.38	1.42	1.56
20	Benzene, ethenyl-	12.4		0.89	0.75	1.26
21	3-Pyridinamine	12.7		9.16	10.54	7.95
22	3-Methyl-pyridine	13.6		4.51	5.43	1.82
23	Benzene, butyl-	14.6				0.70
24	1,4-Benzenediamine	14.8		2.39	4.19	0.52
25	Pyrazine, 2,6-dimethyl-	15.1		1.58	3.66	
26	Pyrazine, ethyl-	15.3			3.78	1.32
27	Pyrazine, 2,3-dimethyl-	15.8		2.08	1.93	
28	1-Hexanol	16.3	5.88			
29	Pyrazine, 2-ethyl-6-methyl-	17.4		1.65	2.39	1.11
30	Pyrazine, 2-ethyl-5-methyl-	17.6		1.23	1.23	0.37
31	2,6-Dimethyl-5-aminopyridine	18.1		6.19	7.60	1.56
32	Benzene, pentyl-	18.6				0.42
33	4-Isopropylpyridine	19.0			0.29	
34	Pyrazine, 2,6-diethyl-	19.4			0.39	
35	Benzene, 1-methyl-2-(2-propenyl)-	19.6			0.49	
36	Pyrazine, 2,6-diethyl-	19.8		4.04	7.03	0.74
37	1-Octen-3-ol	20.3	0.80			
38	Pyrazine, 2,6-diethyl-	20.4		2.37	3.70	
39	2-Furan-carboxaldehyde	20.9		7.03	6.33	3.97
40	Pyrazine, 3,5-diethyl-2-methyl-	21.8			1.24	
41	Butyl aldoxime, 2-methyl-, anti-	22.1			1.50	
42	Ethanone, 1-(2-furanyl)-	22.4				1.31
43	Pyrazine, 3,5-diethyl-2-methyl-	22.5			0.58	
44	Pentaleno[1,2-b]oxirene, octahydro-, (1aα,1bβ,4aα,5aα)-	22.9		3.40		
45	1H-Pyrrole, 3-methyl-	24.5				0.49
46	2-Furancarboxaldehyde,					
5-methyl-	25.1		1.60		2.62	
47	2-Furancarboxylic acid, hydrazide	25.2				0.88
48	1,3,6,10-Dodecatetraene, 3,7,11-trimethyl-, (*Z,E*)-	25.4	1.46	0.74	0.67	
49	5H-5-Methyl-6,7-dihydrocyclopentapyrazine	26.5			0.47	0.32
50	1H-Pyrrole-2-carboxaldehyde, 1-methyl-	26.8			0.49	
51	Cyclohexanol, 5-methyl-2-(1-methylethyl)-, (1α,2β,5α)-	27.6		0.61	1.23	0.69
52	2-Furanmethanol	28.6			0.99	3.26
53	1,6,10-Dodecatrien-3-ol, 3,7,11-trimethyl-	29.1	0.83			
54	*cis*-β-Farnesene	29.1			0.95	
55	(1S,5S)-2-Methyl-5-((R)-6-methylhept-5-en-2-yl)bicyclo[3.1.0]hex-2-ene	30.5	4.41	1.84	0.87	
56	Benzene, 1-(1,5-dimethyl-4-hexenyl)-4-methyl-	32.4	2.85	0.89	0.88	0.59
57	1H-Pyrrole, 1-(2-furanylmethyl)-	34.5			0.18	0.26
58	4(1H)-Pyridinone	36.1		3.63	3.82	1.70
59	Butylated Hydroxytoluene	37.4	4.18			0.17
60	Benzene, 1-isocyano-2-methyl-	37.7		0.49		
61	Ethanone, 1-(1H-pyrrol-2-yl)-	39.3		0.42	0.34	0.25
62	Biphenyl	39.5	0.71			0.18
63	Benzene, 1,1'-oxybis-	40.3	1.61			0.50
64	Phenol	40.7			0.29	
65	1H-Pyrrole-2-carboxaldehyde	41.0		0.37	0.41	0.25

^a^R.T. = retention time of the volatile compound.

^b^Solvent peak is excluded.
