# Supplementary material for: Roasting and Cryogenic Grinding Enhance the Antioxidant Property of Sword Beans (Canavalia gladiata)
Source: J Microbiol Biotechnol. 2020 Aug 21;30(11):1706–19. doi: 10.4014/jmb.2003.03069 (PMC9728382; doi:10.4014/jmb.2003.03069)
Supplement: Supplementary file 1 [file JMB-30-11-1706-supple.pdf]

J. Microbiol. Biotechnol.
https://doi.org/10.4014/jmb.2003.03069

SP1. Characteristics of ^{1}H NMR signals observable in the zone 1-10 ppm of cryogenic-ground raw sword bean

No.	δ (ppm)	Multiplicity	Compound	J (Hz)
1	0.94-0.97	dd	Leucine	14.3
2	0.99; 1.04; 2.27	d; d; q	Valine	7.0; 18.1
3	1.01	d	Isoleucine	7.0
4	1.18	t	Ethanol	14.2
5	1.33	d	Lactate	6.6
6	1.48	d	Alanine	7.2
7	1.91; 2.31; 3.02	t	γ-Aminobutyrate (GABA)	14.9
8	1.93	s	Acetate	-
9	2.14	s	Methionine	-
10	2.45	s	Succinate	-
11	2.60-2.74		Citrate	-
12	2.85-2.97		Asparagine	-
13	3.13	s	Malonate	-
14	3.20	s	Choline	-
15	4.44; 8.08; 8.84; 9.13	s; m; t; s	Trigonelline	-
16	4.59; 5.27	d	Galactose	7.9; 3.8
17	4.66	d	Glucose	8.0
18	5.42	d	Sucrose	3.9
19	5.81; 7.53	d	Uracil	8.5
20	6.90; 7.19	d	Tyrosine	8.6

21	7.3-7.4	m	Phenylalanine	-
22	8.46	s	Formate	-

SP2. Concentration for some metabolites in cryogenic-ground sword bean determined by ^{1}H NMR signals

Position	Compound	Concentration (mM)			
		Raw bean	Light roasting	Medium roasting	Dark roasting
1	Leucine	0.73 ± 0.01	ND*	ND	ND
2	Valine	0.98 ± 0.01	0.10 ± 0.01	0.12 ± 0.01	ND
3	Isoleucine	0.51 ± 0.01	ND	ND	ND
4	Ethanol	2.30 ± 0.21	0.16 ± 0.02	ND	ND
5	Lactate	1.58 ± 0.02	0.31 ± 0.03	0.15 ± 0.01	0.28 ± 0.02
6	Alanine	1.10 ± 0.07	0.23 ± 0.02	0.17 ± 0.01	ND
7	GABA	2.34 ± 0.16	ND	ND	ND
8	Acetate	2.79 ± 0.00	1.32 ± 0.11	1.01 ± 0.06	1.05 ± 0.12
9	Methionine	0.34 ± 0.00	ND	ND	ND
10	Succinate	0.45 ± 0.00	ND	ND	ND
11	Citrate	1.01 ± 0.00	0.92 ± 0.07	0.60 ± 0.08	0.25 ± 0.02
12	Asparagine	1.01 ± 0.00	ND	ND	ND
13	Malonate	1.54 ± 0.01	0.96 ± 0.08	0.60 ± 0.04	0.47 ± 0.04
14	Choline	1.31 ± 0.01	0.72 ± 0.03	0.74 ± 0.05	0.66 ± 0.08
15	Trigonelline	0.66 ± 0.06	0.54 ± 0.01	0.32 ± 0.03	0.24 ± 0.03
16	Galactose	2.74 ± 0.11	ND	ND	ND
17	Glucose	4.22 ± 0.10	1.06 ± 0.07	ND	ND
18	Sucrose	4.58 ± 0.02	6.35 ± 0.16	4.55 ± 0.26	0.54 ± 0.02
19	Uracil	0.34 ± 0.01	ND	ND	ND

20	Tyrosine	0.41 ± 0.01	ND	ND	ND
21	Phenylalanine	0.31 ± 0.01	ND	ND	ND
22	Formate	3.14 ± 0.00	ND	ND	ND
23	2-Furoate	ND	ND	ND	0.17 ± 0.02

All processes were done 3 times repeatedly. Values are mean ± SD (n=3)

* Not detected
